# From Grafts to Human Bioengineered Vascularized Skin Substitutes

**DOI:** 10.3390/ijms21218197

**Published:** 2020-11-02

**Authors:** Wasima Oualla-Bachiri, Ana Fernández-González, María I. Quiñones-Vico, Salvador Arias-Santiago

**Affiliations:** 1 Cell Production and Tissue Engineering Unit, Virgen de las Nieves University Hospital, 18014 Granada, Spain; wasimaouallabachiri@gmail.com (W.O.-B.); mariai.quinones@juntadeandalucia.es (M.I.Q.-V.); salvadorarias@ugr.es (S.A.-S.); 2 Biosanitary Institute of Granada (ibs. GRANADA), 18014 Granada, Spain; 3 Andalusian Network of Design and Translation of Advanced Therapies, 41092 Sevilla, Spain; 4 Dermatology Department, Virgen de las Nieves University Hospital, 18014 Granada, Spain; 5 Dermatology Department, School of Medicine, Granada University, 18016 Granada, Spain

**Keywords:** angiogenesis, endothelial cells, grafts, skin substitutes, tissue engineering, trilayered, vascularization, wound healing

## Abstract

The skin plays an important role in the maintenance of the human’s body physiological homeostasis. It acts as a coverage that protects against infective microorganism or biomechanical impacts. Skin is also implied in thermal regulation and fluid balance. However, skin can suffer several damages that impede normal wound-healing responses and lead to chronic wounds. Since the use of autografts, allografts, and xenografts present source limitations and intense rejection associated problems, bioengineered artificial skin substitutes (BASS) have emerged as a promising solution to address these problems. Despite this, currently available skin substitutes have many drawbacks, and an ideal skin substitute has not been developed yet. The advances that have been produced on tissue engineering techniques have enabled improving and developing new arising skin substitutes. The aim of this review is to outline these advances, including commercially available skin substitutes, to finally focus on future tissue engineering perspectives leading to the creation of autologous prevascularized skin equivalents with a hypodermal-like layer to achieve an exemplary skin substitute that fulfills all the biological characteristics of native skin and contributes to wound healing.

## 1. Introduction

Skin is the greatest organ of the human anatomy. It constitutes a protective barrier that isolates our body from harmful agents and injuries. The skin structure consists of three differentiated layers, which are the epidermis, dermis, and hypodermis. Each one has its own characteristics such as physical properties or cell composition. Epidermis is the most superficial layer, and it is composed principally of keratinocytes. Dermis, in the middle, is comprised mainly of fibroblasts, while it also has a considerable quantity of endothelial cells (ECs) that form dermal vascular networks. On the hypodermis, the inner layer, adipocytes, and EC can be found. 

Our skin has the ability to self-renew. However, it can suffer several damages such as burns or deep wounds that cannot regenerate on their own. In addition, several skin injuries are associated with large and broad clinical problems that can end in morbidities. Around more than 11 million people are affected by burn injuries worldwide, and the prevalence of infection in burn units is 66% [1].

Skin grafts have been used for a long time as a solution to cover wounds; however, it is not even possible to dress massive wounds with grafts. The location of the injury, the patient state, the difficult of having a healthy donor site, and multiple factors make this approach impossible. So, there has been a real need to research another efficient way to treat these patients.

Tissue engineering [2,3] is an emergent field that is constantly evolving to provide clinical solutions for patients who need tissue replacement. A large amount of tissues has been recreated under laboratory conditions, coming to create complete organs. However, the first tissue-engineered organ that went from preclinical research to clinical application was the skin [4].

Skin bioengineered substitutes emerged as new advanced therapies to minimize all the problems that are associated with skin transplantation. These substitutes are developed in vitro to recreate human skin. Apart from being employed as grafts for the renewal of missing skin, they can be used also for other applications such as in vitro human skin models development for diseases or for testing pharmaceutical products.

Many skin substitutes for wound cover are commercially available, while a large amount of them are at the preclinical research stage. They vary on multiple properties such as the number of layers, composition, cost, technique used for its fabrication, etc. Despite the amount of skin substitutes that can be found, they have similar purposes: providing the patient with a barrier, serving as a protection against microorganisms, reducing pain, and promoting wound healing. This last topic is in the point of view of many tissue engineering researchers. Note that the goal is not only covering the wound; a skin substitute that enhances the healing process to provide wellness to the patient has to be created. Obtaining a full-layer vascularized skin substitute could accelerate wound heal and ameliorate the quality of life of burn patients. For example, children and adolescents are more susceptible to hypertrophic scarring during growth [5], so a vascularized skin substitute could be a suitable approach for a complete and effective wound heal. In addition, healing capability is affected by aging, because of a decrease in skin elasticity and strength and a reduction in blood flow to the extremities [6], so a prevascularized substitute could mitigate the healing process, especially in these cases.

Despite all the achievements that have been made on this area of research, an ideal skin substitute does not exist yet. Not all features of native skin have been recreated on an in vitro engineered skin substitute [7].

The objective of this review is to collect the progress that has been made in human skin substitutes engineering in the last years, to finally focus on trilayer vascularized skin substitutes, because they could be considered as the fullest solution until now for damaged skin replacement.

## 2. Chronological Review

The original report of using skin grafts dates back to 2500 BC, when they were used in India to treat serious injuries located on the extremities [8]. It was called the “Ancient Indian Method”. However, up to the beginning of the nineteenth century, nothing of importance was done in regard to skin grafts. The controversy that sparked Reverdin in 1869 with the report that he presented to the Société Impériale de Chirurgie in Paris, about the healing of granulating wounds with small bits of skin that he called “epidermic grafts”, and its discussion, started the flame of interest in the subject. Finally, he admitted that his graft had a portion of corium, so it was not an epidermic graft.

In the 19th century, skin grafting experienced an important technological evolution. Xenografts were developed in 1804, and the use of allografts instead of autologous skin grafts emerged in the 1870s with Thiersch. His method of taking large films of epidermis with a thin portion of dermis which was presented at the Fifteenth Congress of the German Surgical Association in 1886. Ollier did the same thing 12 years previously, but Thiersch gave no credit to him when he presented his method [8].

Although the use of autologous skin grafts has numerous advantages, they were not able to replace substantial skin loss, and allogenic skin grafts carried a risk of rejection. Other alternatives were emerging in response to these drawbacks.

Synthetic grafts were first described in 1880, and Mangoldt invented the “epithelial cell seeding” concept in 1895 [9]. It was the beginning of a new research area. When Rheinwald and Green cultured epidermis from a patient’s own cells in 1975 [10], they did not know that they were setting a precedent in the tissue engineering field [4]. It was a revolutionary milestone and it has fostered intense research in this area since then. In 1981, O´Conner et al. created autologous sheets of cultured epithelium from keratinocytes cell expansion from two burn patients. These epithelial sheets were used to cover their extensive full thickness, becoming the first grafting of autologous epithelium on extensive burn patients with success around the world [11,12].

These cultured epidermal autografts (CEAs) were also used with success on two pediatric patients [13]. Unfortunately, a sheet conformed only by keratinocytes is very fragile, and it is not always possible to use it in the clinical practice.

Burke et al. generated a dermal substitute in 1981, and they used it to treat ten patients with extensive burn injuries [14]. That artificial dermis was used on major burns by Heimbach et al., in a randomized multicentric clinical trial, and it is currently known as an Integra™ Dermal Regeneration Template. Cuono et al., by the mid-1980s, demonstrated the importance of having a dermal vascularized component as a bed for CEA having his own method for its preparation [15]. The 1990s was the decade where composite skin substitutes appeared, starting from Cuono’s technique to integrate both a dermal and an epidermal layer in one substitute. Several research centers with fairly reproducible success adopted his strategy since that decade [16,17]. 

These events caused the birth of what Langer and Vacanti described in 1993 [2] with the term “tissue engineering”. Since then, a large range of skin substitutes have been manufactured. From single-layer substitutes to artificial skins or genetically modified substitutes and so on can be found. Each substitute has its own singularity, and today´s research in this field is focusing on the in vitro creation of personalized substitutes that avoid the risk of rejection and allow the transplantation of any surface needed without limitations.

**Figure 1:**
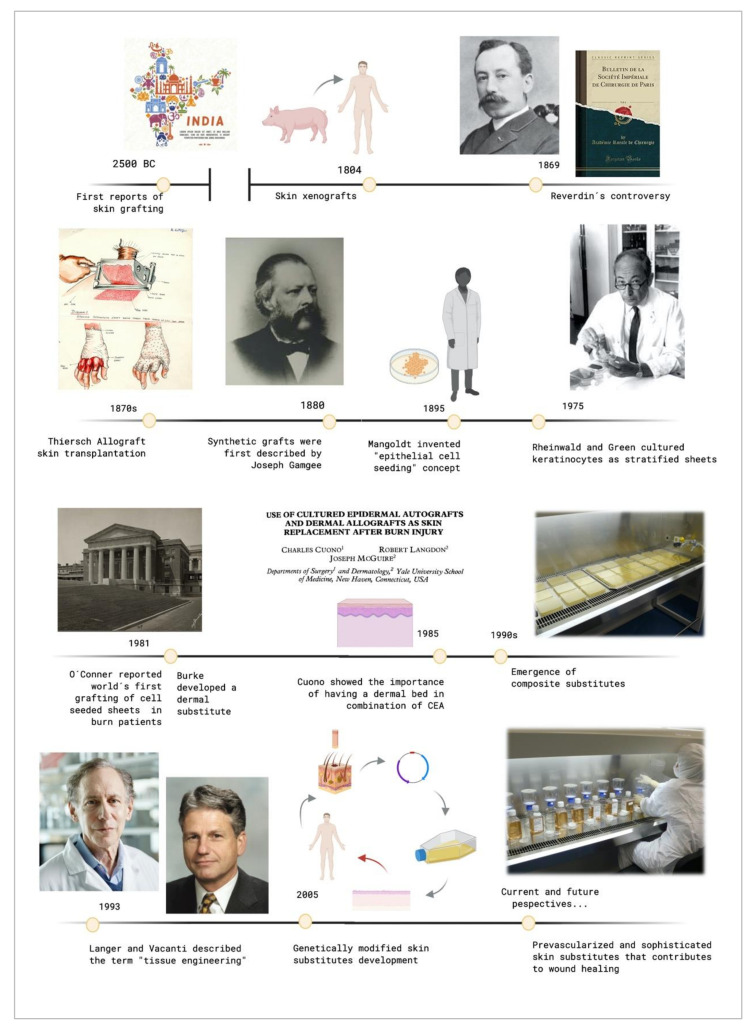
Timeline of the crucial events that contributed to tissue engineering development. Created with BioRender.com.

Figure 1 shows the evolutive timeline of the events described above.

## 3. Clinical Demands for Bioengineered Artificial Skin Substitutes (BASS)

Bioengineered skin equivalents offer promising perspectives. However, they have to fulfill a series of characteristics that allows its clinical use. 

### 3.1. Ethical and Legal Requirements

Before their use on patients or commercialization, clinical trials using skin substitutes have to been done. To be accepted, these clinical trials have to conform the Declaration of Helsinki and the guidelines of Good Clinical Practice (GCP). Moreover, cell-based therapies encounter some limitations as they are considered “Advanced Therapy Medicinal Products” (ATMPs), which applies the same good manufacturing practice (GMP) procedures as for pharmaceutical products and biological drug development [18]. In Europe, these therapies have to adhere to the European Medicines Agency (EMA) guidelines for ATMPs. ATMPs are divided into four categories: somatic cell therapy medicinal products, tissue-engineered products, gene therapy medicinal products, and the combined ATMPs. ATMPs in Europe are governed by Directive 2009/120/EC and Regulation 1394/2007/EC [19,20]. In the United States, advanced therapies are regulated as biologic products by the Food and Drug Administration (FDA, the federal regulatory medicines agency). ATMPs in the United States can be sub-classified into two groups: gene therapy and cellular therapy products. Biological products, and therefore advanced therapies, are regulated under section 351 of the Public Health Services Act (PHSA) and under the Federal Food, Drug, and Cosmetic Act (FDCA) [21]. However, Japan, Australia, and Canada have a similar regulatory framework than in Europe [22,23].

An ethical committee has to approve the clinical trial before its start, and an Institutional Review Board (IRB) has to review and approve all the procedures. All patients have to be given detailed information about the study. An informed consent from each participant on the clinical trial for each procedure has to be obtained. 

However, in some cases, the patient state is very serious, and other therapies have not made any effect, so hospital exemptions are need for “compassionate use” to assure a patient´s safety. Article 37 of the Declaration of Helsinki shows this possibility.

‘‘In the treatment of an individual patient, where proven interventions do not exist or other known interventions have been ineffective, the physician, after seeking expert advice, with informed consent from the patient or a legally authorized representative, may use an unproven intervention if in the physician’s judgement it offers hope of saving life, reestablishing health, or alleviating suffering. This intervention should subsequently be made the object of research, designed to evaluate its safety and efficacy. In all cases, new information must be recorded and, where appropriate, made publicly available.’’ 

Apart from legal and ethical requirements, a skin substitute has to have the following characteristics.

### 3.2. Protective Functions and Resistance to Infection

Skin substitutes must be sterile and able to protect the wound from infectious agents such as bacteria, parasites, or fungus mainly on chronic wounds or burns [24,25]. Serial clinical problems such as sepsis and systemic inflammation could derive from a microbial infection. Prior to its implantation to the patient, there has to be evidence that the product is suitable and secure for its clinical application. 

Wound care is an important feature that has to be considered. This can be accomplished by topical wound coverings with povidone–iodine solutions or cotton gauze dressings. However, it may not be efficient on patients who have some comorbidities that affect the correct care of the wound [26]. 

### 3.3. Biological Functions

Cells that compose the skin substitute should be capable of proliferating and differentiating in a similar manner as it happens on their natural environment, in addition to having proper functions according to the physiological desired effect. Skin substitutes must have rheology comparable to the native skin and they should be biocompatible and biodegradable. The use of non-biological components can present biocompatibility difficulties [27]. 

### 3.4. Able to Prevent Water Loss and Water Accumulation

A skin substitute has to transmit water in a similar way to the normal skin, avoiding fluid loss and accumulation [28]. It is a critical point because water loss [29] and the risk of infection are the main problems that can be encountered in full-thickness wounds. Many burned patients die because of dehydration. However, an excessive accumulation of water could promote infection.

### 3.5. Adhesiveness

The adhesiveness of the skin equivalent depends on being a temporary substitute or a permanent substitute. When using temporary skin substitutes, they have to be easy to remove because they are used only as a first cover of the wound. If the skin equivalents are permanent substitutes, they have to stick on the wound. The rapid adhesion to the wound surface is an essential feature for cell differentiation [30], especially on those substitutes that are cell-based.

### 3.6. Low Antigenicity 

The principal problem of allogenic and xenogeneic scaffolds is the risk of rejection. An ideal skin substitute has to prevent the host refusal response. For example, burned patients are frequently in an unstable situation and their immune system is compromised, so the skin substitutes used for their treatment cannot deteriorate their situation by triggering an immunogenic response.

### 3.7. Conform to Irregular Wound Surfaces

In some cases, the wound is not in a regular site. The graft should be pliable to be simply placed on surfaces that can be uneven such as fingers or elbows. Skin substitutes have to be manageable and adaptable but not fragile for avoiding scarring.

### 3.8. Withstand Shear Forces and Mechanical Tensions

Skin substitutes are manufactured to hold shear forces and mechanical tensions [7,31] that are applied during the substitute placement. The graft also has to be flexible and robust enough to be surgically handled. 

### 3.9. Hypoxia Tolerant

In a burn wound, tissue is not well oxygenized, so the substitute must tolerate low oxygen concentrations. This event is specially intensified on cell-based substitutes. 

### 3.10. Dermal and Epidermal Components

As seen in the *chronological review*, a combination of dermal and epidermal components is needed to make an effective skin substitute [32,33,34]. Cuono and his colleagues demonstrated the fact that it is essential to have a dermal layer as a bed to put on the epidermal layer [15].

### 3.11. Easy to Prepare

The skin substitute´s preparation should be easy. This would reduce costs and permit large-scale fabrication to guarantee broad availability. An optimization of fabrication procedures would speed up the process.

### 3.12. Long Shelf Life and Easily Storage

As the costs of skin substitutes are expected to rise, they should have a long shelf life and easy storage. Greater storage requisites would only increase the skin substitutes’ price. 

### 3.13. Suitable Cost/Effectiveness

All the therapies that can save the patient´s life should have reduced costs to become accessible for all the patients that need them [35]. Skin equivalents fit into this category, especially when they are used on large total body surface area (TBSA) burnt patients.

## 4. Skin Substitutes Classification

**Figure 2:**
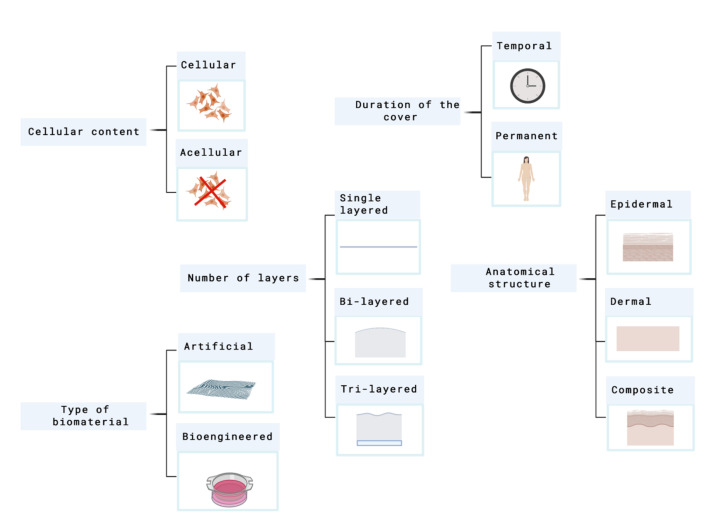
Principal parameters used to classify skin substitutes. Created with BioRender.com.

Skin substitutes classification depends on the issue in focus. As many factors have to be taken into account, skin substitutes classification has been conflicting and overlapping in many cases. Figure 2 shows the principal parameters that are used to classify skin substitutes.

In 2001, Balasubramani and their colleagues [36], proposed a classification where they put the skin substitutes into three categories attending to the next criteria:

I. Cultured epidermal substitutes
II. Dermal components coming from skin or extracellular matrix (ECM) components
III. Substitutes that include both dermal and epidermal components.

This system does not differentiate between cellular and acellular components and does not include dermal constructs fabricated from synthetic polymers or dermal substitutes such as Integra^®^. 

Balasubramani´s classification was replaced by Kumar´s system, which was published in 2008. Currently, it is considered the most frequently used classification system in this field [37]. 

**Figure 3:**
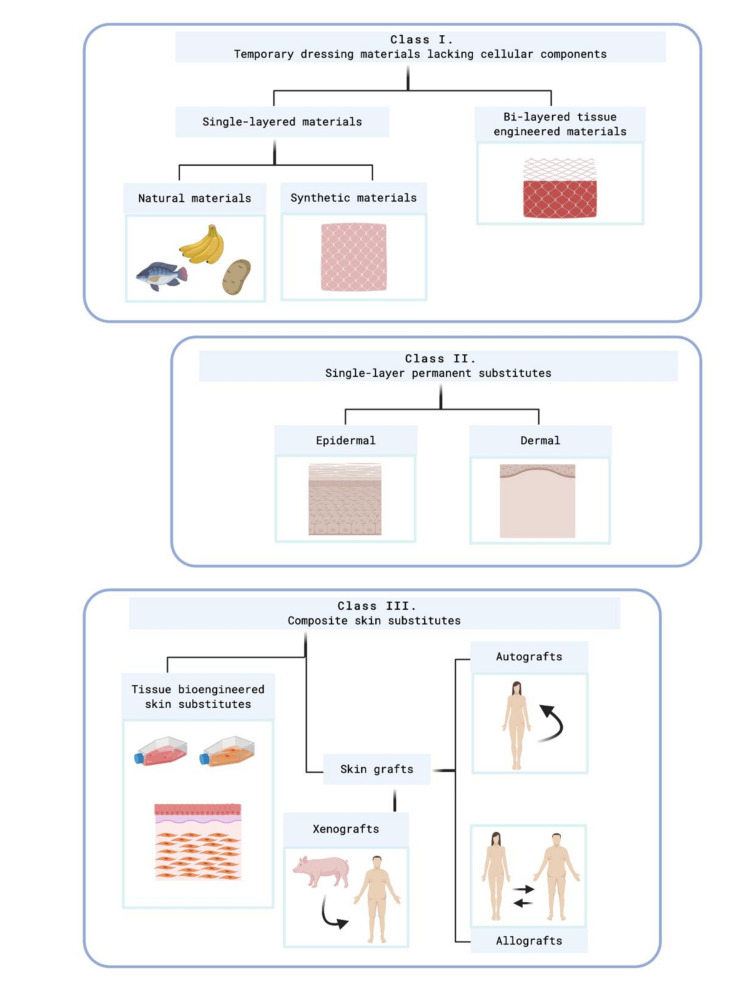
Kumar’s classification system. Created with BioRender.com. At class II, subclass epidermal, Kumar includes cultured epidermal autografts (CEA) and Apligraf^®^, which are cellular skin substitutes. At class II, subclass epidermal, acelular skin substitutes which are made of extracellular matrix (ECM) compounds are included as PermacolTM^®^, Matriderm^®^, Alloderm^®^, or Kollagen^®^.

Figure 3 shows this classification.

However, it is necessary to highlight that a standard classification system does not exist. The parameters showed above have similar importance so, finally, each author could classify the skin equivalents under its criteria. 

### Temporary Skin Substitutes

Temporary skin substitutes play a crucial role in the process of wound healing because they allow skin regeneration while the permanent skin substitute is not implanted yet. They are used as a first dress on deep injuries. When the wound is covered, infection risks are reduced, which indirectly also reduces corrective surgeries and hospitalization costs [18].

Conventional temporary skin substitutes are xenogeneic decellularized skin and allogenic cadaveric human skin. Porcine xenografts are probably the most frequently used temporary skin covering worldwide, which is attributable to its easy accessibility and storage [38]. Allogenic skin equivalents that comes from human cadaveric skin become engrafted to the wound site between 14 and 21 days, and when it is taken out at surgery, it leaves the dermal components as a viable bed for CEA [17]. However, the main drawbacks of conventional substitutes are rejection-related risks. Other temporary skin substitutes are synthetic substitutes, human amniotic membrane, and other natural skins. Table 1 summarizes different types of temporary skin substitutes.

Synthetic substitutes are principally composed of hydrogels and hydrofibers [39]. Despite these synthetic dressings acting as a cover capable of protecting skin injury, they do not contribute to skin tissue regeneration, especially in the case of patients who present extensive wounds. 

The human amniotic membrane (HAM), the innermost layer in the fetal placenta, is another element that has been used as a successful biological skin substitute in wounds. It has been shown that it accelerates wound healing, prevents infection, and alleviates pain [40]. HAM is a special tissue with anti-inflammatory and anti-fibrotic properties [41]. Amniotic epithelial cells (AECs) and amniotic mesenchymal cells (AMCs) compose the HAM. Both kind of cells have self-renewal properties and the capability to differentiate to multiple lineage [42]. 

Other alternative natural skins such as banana leaves [43], potato peel [40], or tilapia fish skin [44] are used as first covers in developing countries where more sophisticated therapies are off-limits to the population. 

As temporary substitutes are “temporal solutions”, a permanent skin substitute must dress the injury to replace the missed skin. The category of permanent skin substitutes includes the grafting of cultured skin cells with or without the support of a scaffold. There are many skin substitutes that are still in the research stage; however, a broad range of commercial skin substitutes can be encountered on the market.

## 5. Commercially Available Skin Substitutes

Since the research of human skin substitutes to cover deep wounds was started, many commercially substitutes have arisen. The first commercially available substitute approved by the FDA was Epicel^®^, and a broad range of skin substitutes have emerged since then. From spray-applied epidermal equivalents to composite scaffolds that include ECM compounds, all them are present on the market. Before remarking on a few relevant characteristics of some commercial skin equivalents, they are going to be divided according to their cellular content. The cellularity is a relevant fact that determines the biological analogy of the substitute with the skin. However, some restrictions are applied to their use. No cellular commercial substitutes have been approved for use on humans in Europe, except for clinical trials or compassionate use.

Cellular substitutes include the following. Apligraf^®^ was the first skin substitute that was approved by the FDA to stimulate healing in ulcers because of its capability of producing cytokines and growth factors mimicking the native skin [45]. Clinical trials data support that Bioseed-S^®^ has almost 50% more efficacy when compared with conventional treatment for chronic venous leg ulcers (VLUs) [46]. However, there is no information relative to its use in burned patients. CryoSkin^®^ is a cell spray prepared upon clinician’s request [47]. Dermagraft^®^ delivers a dermal matrix that is collagen-rich to a prepared ulcer wound bed. Its fibroblasts maintain the capacity for secreting a diversity of regulatory and structural proteins due to their metabolically activity [48,49]. EpidexTM^®^ is efficient for split-thickness skin autografting by promoting the healing and following up the closure of recalcitrant vascular leg ulcers (VLUs) [50]. EPIBASE^®^ and Recell^®^ are made of suspensions of a patient´s cells that are sprayed on the wound [51,52]. Hyalograft 3D^®^ in combination with Laserskin^®^ (TissueTech Autograft System) conformed the first full-thickness autologous substitute [53]. OrCel^®^ exhibits reduced scarring [54]. Transcyte^®^ promotes a faster epithelial renewal, while it requires fewer dressing changes and autografting procedures. However, as it has foreskin fibroblasts, there is a risk of inflammation and rejection. However, as it has foreskin fibroblasts, there is a risk of inflammation and rejection [55]. 

In the acellular group, the following substitutes are included. Alloderm^®^ has been widely used for reconstruction after abdominal or breast surgery. It has been reported that it enhances vascularization when used in soft tissues such as the abdominal wall [56]. Biobrane^®^ minimizes bacteria proliferation by minimizing dead space, controlling vapor loss, and allowing conformability to surface irregularities, owing to its flexibility. It becomes detached after epithelization of the wound. However, it is not universally accepted because it has porcine origin, risk of infection, and high costs [57,58]. Integra^®^, even being an acellular substitute, has been utilized in combination with Recell^®^ cell suspensions in a one-step procedure [59]. Matriderm^®^, because of its hemostatic features, decreases the risk of hematoma after skin grafting [60]. In OASIS^®^, unlike other purified collagen wound care products, ECM components are retained in their bioactive forms [61,62]. Permacol™ crosslinking offers protection from host collagenase biologic degradation [63]. Suprathel^®^ is not very efficient in full thickness wound healing, although it has powerful anti-sepsis properties and it can stall bleeding efficiently [64,65]. Terudermis^®^, despite being a dermal substitute made of collagen and sillicone, when loaded with cultured fibroblasts, EC, platelet-derived growth factor, and then applied to rodent in vivo models, it showed not only angiogenesis achievement but also the possibility of using the equivalent simultaneously with a split-thickness skin grafts (STSG) for a one-step operative procedure [66].

Table 2 gives details of the commercially available skin substitutes mentioned above. 

Commercially available skin substitutes have several limitations. Allogenic and xenogeneic substitutes can have associated rejection problems. Specifically, in xenografts, commonly from pig skin, rejection is due to the exposition of the patient to antigens expressed on the vascular EC of the pig, mainly galactose-α1,3-galactose. The human body has performed xenoreactive antibodies that bind to these antigens and activate, resulting in endothelium damage and thrombosis [78]. Some strategies to reduce or prevent the rejection include the development of genetically engineered pigs by knocking out specific genes, for example, the α1,3-galactosyltransferase gene [78,79]. Allograft rejection is mediated by the activation of T cells [79,80], which can specifically destroy the major histocompatibility complex (MHC) of incompatible cells. This activation is mediated by the epidermal Langerhans cells of the allogenic skin, which migrate to the draining lymph node of the patient to activate the T cells; it is also activated by dendritic cells (DCs) of the dermis, and it can be achieved by a complex mechanism involving three different pathways [80]. The direct activation pathway consists in the T-cells recognition of the donor intact MHC antigens on donor DCs. In an indirect activation pathway, T cells recognize processed donor antigens presented by self-MHC molecules on host DCs. Lastly, some studies have shown that T cells can become activated by the recognition of donor MHC molecules transferred on recipient DCs in a semidirect pathway. It should be remarked that the activation of T cells via direct or indirect pathways is sufficient to trigger the acute rejection of allografts, while the contribution of the semidirect pathway is still unknown. B cells and natural killer (NK) cells seem to play a role in the allograft rejection process as well [80]. B cells present donor antigens to T cells by the opsonization of donor cells and by direct complement-dependent antibody-mediated cytotoxicity. NK cells can directly or indirectly kill donor cells, although these mechanisms are poorly understood. 

Thus, the allograft can be treated with agents that suppress the function of Langerhans cells and DCs before its implantation on the patient. This approach can slow down the rejection process [78,79]. 

Cellular constructs do not achieve completely biological functions. It has been reported that an inefficient vascularization exists when xenogeneic substitutes are used [81,82]. In addition, many Muslim patients reject the use of skin substitutes that have porcine origin such as Biobrane^®^. Allogenic sources are limited. In addition, the majority of composite skin substitutes present on the market are mostly composed of a biocompatible polymeric scaffold with one or more types of skin cells growing on it [83]. Since these skin equivalents include cultured cells, they take a long manufacturing time, request careful handling under aseptic conditions, and have brief shelf lives [84]. 

What is more, skin substitutes up until now majorly focused on designing natural, artificial, or mixed dermal and epidermal components. In case of full thickness wound healing, all the three layers of skin need to be regenerated, which can occasionally include muscular tissue as well [83]. However, a commercially available skin substitute whose structure mimics the human rheology of the three skin layers, epidermis, dermis, and hypodermis, has not been already fabricated. Even more, a prevascularized skin equivalent on the market have not be found. It is impacting if it is considered that current available skin substitutes lack a layer that simulates hypodermis, considering the importance of this layer.

## 6. Trilayered Skin Substitutes

The in vitro engineering skin substitutes have significantly progressed [85]. However, currently available skin substitutes lack an inner fat layer that would functionally contribute to some properties of normal skin by mimicking the hypodermal layer [86]. 

Even so, most of the now called “trilayered substitutes” have a third layer apart from dermis and epidermis that is not hypodermis. If “trilayer skin substitutes” is searched in most popular literature databases, trilayer substitutes that contain a hypodermal layer are not found. Most of the trilayer substitutes found were synthetic trilayered matrixes or dermoepidermal substitutes with a synthetic third layer that “mimics” hypodermis or conforms another functional layer such as the basal membrane.

Haldar et al. created a bioengineered fine trilayer skin tissue equivalent for efficient deep wound healing [83]. However, the third layer, which could be considered as a “hypodermal layer”, was made of gelatin, and there were no cellular components such as adipocytes or EC. 

There are also some publications of three-layer substitutes where the third layer is a thin film that mimics the basement membrane. Many bi-layered scaffolds have been created to be seeded with keratinocytes on a layer and fibroblasts on the other layer. However, as fibroblasts proliferate faster than keratinocytes, without a physical separation, fibroblasts could outgrow keratinocytes, so a structure that imitates the basement membrane would be needed. An example of this is the biomimetic basement membrane substitute based on trilayered nanofibrous scaffolds developed by Huang et al. [87]. Also Lin et al. developed a “trilayer” scaffold which was composed of dermis, epidermis and basement membrane [88]. Their scaffold was chitosan-based with nanofibers simulating epidermis and achieving a more precise replication of the trilayered structure of a full-thickness skin than a single or bi-layered scaffold.

**Figure 4:**
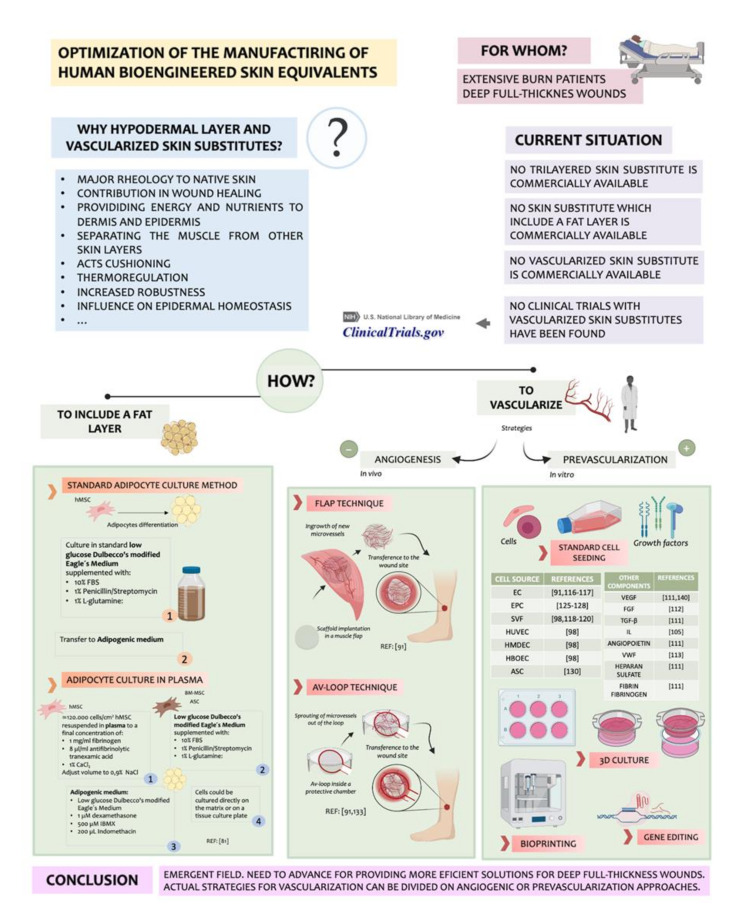
Synthetization of the strategies used for vascularizing skin substitutes.

Attending to what was found in the literature, it can be intuited that full-layer skin substitutes that contain a hypodermal layer are not yet well established in the panorama of tissue engineering. This kind of skin substitute will be useful specially in the case of deep wounds where the subcutaneous layer is lost. On the basis of this, the importance of this layer and the necessity of including it on future emerging bioengineered skin substitutes is going to be analyzed. Figure 4 summarizes this point.

The hypodermis is the inner layer of the skin. It is conformed principally of adipocytes and EC. It provides energy and nutrients through microvascular capillaries to the dermis and epidermis, having a crucial role in vascularization. It also isolates and separates physically the muscle from the other skin layers. In addition, as a fat layer, it facilities the mobility and provides cushioning. Other functions of hypodermis are thermoregulation, sensorial, immunity, and endocrine. 

For a long time, researchers have ignored the influence of hypodermis on epidermal homeostasis. Skin substitutes that are currently used in the clinic do not achieve yet the functions of native skin because they do not reproduce all its structure. Even more, a trilayered bioengineered skin substitute would have an increased robustness because of the presence of three physical layers. In addition, its functionality would improve the esthetic appearance of cultured skin grafts after the placement on the patient. Despite the advantages mentioned above, few studies about trilayer skin substitutes including an adipocyte-containing hypodermis have been reported. Trottier et al., in 2008 [89], was one of the first authors to report this kind of skin substitute with Vermette [90]. Unfortunately, they showed limited information about the skin equivalent. 

Monfort and her collaborators created in 2012 a skin substitute that had a hypodermis. To create it, they cultured bone marrow mesenchymal stem cells (BM-MSC) and adipose tissue-derived stromal cells (ASC) in human plasma. They also added adipogenic clues for differentiating the adipocytes to create the plasma-based hypodermal layer. The presence of these cells on the construct contributed to the epidermal differentiation, and it was demonstrated with more levels of keratinocytes proliferation and differentiation. Secreted leptin was also studied, and they reported a robust expression when adipocytes were grown under 3D hydrogel conditions.

### 6.1. Importance of Vascularization in Wound Healing

Vascularization is a key point in the development of skin substitutes, as it finally determines the complete biological function of bioengineered skin implants. If the substitute is not well vascularized, oxygen and nutrients will not be correctly supplied to the wound, which may result in infection or necrosis [91].

To reinforce our theory about why it is so important to advance research on the creation of prevascularized skin substitutes, we need to comprehend also what happens physiologically after a skin injury.

**Figure 5:**
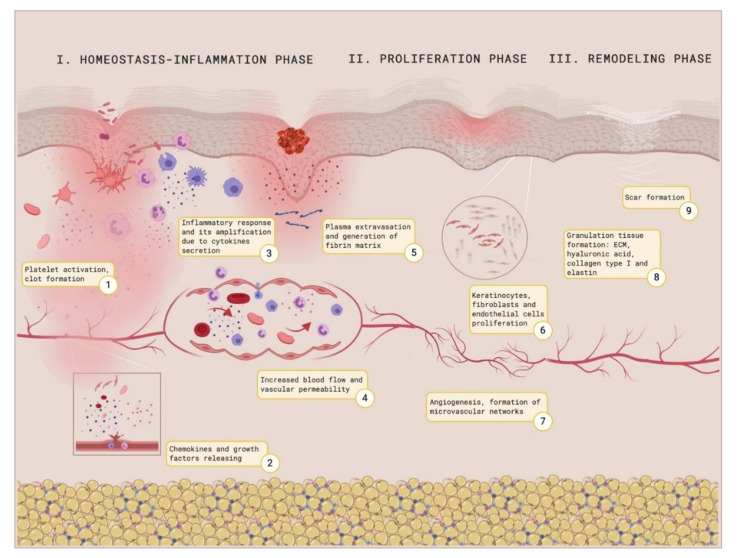
Wound healing illustration. Created with BioRender.

After a skin injury, an integrated healing response to restore the damaged tissue is coordinated sophisticatedly, even though it may result in a complex process. Many cell types and mediators interact sequentially to coordinate the healing response [92]. This response proceeds in three succeeding and overlaying phases that include the hemostasis/inflammatory phase, the proliferation phase, and the remodeling phase [93]. The first step in the healing response is to achieve hemostasis. Surrounding platelets, as a consequence of their interaction with the exposed ECM and especially with collagen and the von Willebrand factor, will adhere to collagen through their glycoprotein receptor complex [94]. This interaction will activate phospholipase A2, which will release arachidonic acid from a phospholipids membrane. Cyclooxygenase, also known as prostaglandin–endoperoxide synthase, will catalyze the conversion of arachidonic acid to prostaglandin H2, which will be the precursor of other prostaglandins and thromboxane. Thromboxane A2 will participate on vasoconstriction and platelet aggregation, which will ease the adhesion of more platelets and the clot formation. These platelets will also participate in the activation of macrophages by the liberation of cytokines [95]. Neutrophils and macrophages secrete pro-inflammatory cytokines and thereby amplify the inflammatory response [96]. Their accumulation on the wound location facilitates the phagocytosis of bacteria and damaged tissue with the purpose of providing a good environment for wound healing [97]. After the inflammatory phase, vessels will dilate to increase the blood supply around the wound, aiming to apport nutrients, growth factors, and cells such as EC, keratinocytes, and fibroblasts. Subsequently, they will proliferate due to the secretion of cytokines and growth factors such as transforming growth factor-β (TGF-β), interleukins (ILs), and proangiogenic factors [98]. Angiogenesis will be initiated by endothelial progenitors, which will derive from the invasion of vascular EC and capillaries to form microvascular networks [99,100,101]. The first clot and the fibrin formed matrix will be replaced by a complete ECM comprised of proteoglycans, elastin, hyaluronic acid, and other components. Finally, the remodeling phase implies a balance between the apoptosis of existing cells and production of new cells. The formation of collagen type I is critical in this phase. Figure 5 shows the process of wound healing.

In a patient with a full-thickness wound, where the skin functions are disrupted, this normal response is incomplete or absent. When non-prevascularized dermo-epidermal skin substitutes are implanted in a patient, they suffer a physiological crisis due to impaired nutrition, oxygen, and growth factor supply between 14 and 21 days after transplantation. The absence of functional vascular structures is still one of central hurdles in tissue engineering and regenerative medicine [102] and nowadays, it could be considered as a critical factor for clinical applications in this field. Although numerous publications treat the topic, skin vascularization has not been well established yet. Many biomaterial-based scaffolds fail due to poor graft take and integration with host tissue [103].

### 6.2. Strategies for Vascularization

Aiming to this, numerous approaches have been proposed to achieve the vascularization of skin grafts [104,105,106]. These strategies can be classified as angiogenic or prevascularization approaches. 

#### 6.2.1. Angiogenic Strategies

Using an angiogenic approach implies the stimulation of blood vessels ingrowth. However, due to the slowness of the process, it is inadequate for the quick vascularization of huge implants [107,108]. 

In vivo strategies include, apart from angiogenic ingrowth, the flap technique and the arteriovenous loop (AV-loop) technique. In the flap technique, a scaffold is placed into a muscle flap to allow the arbitrary ingrowth of newly developing microvessels. After prevascularization, the entire flap with the incorporated implant is freely transferred to the defect site, where the vascular pedicle of the flap is surgically anastomosed to host vessels [108]. The AV-loop technique involves the integration of an AV-loop inside a protective growth chamber to originate a prevascularized tissue construct by the spontaneous ripening of vessels out of the loop. Then, it is transferred and anastomosed surgically to be integrated with the host vessels. This technique, in comparison with the flap technique, allows the generation of prevascularized tissue, which is not embedded in the surrounding muscle tissue [109].

#### 6.2.2. Prevascularization Strategies

The prevascularization approach is a promising strategy that implies the creation of microvascular networks on the substitute before becoming engrafted to the patient. This means a rapid blood supply due to the previous formation of the vessels [110,111]. It has been shown that prevascularized grafts are capable of anastomosing with the host vessel following the transplantation, which carries some advantages as the correct incorporation of the skin substitute with an increase in the average cell survival [112,113,114,115]. It also has been reported that the use of prevascularized skin substitutes derives on a rapid perfusion that results in an efficient blood supply to the wound, an increased collagen type I deposition, an increased cell proliferation of both dermis and epidermis, and a decreased expression of wound healing markers, which is probably due to the reach of epidermal homeostasis and reduced contraction [116].

Different cell types may be suitable for the vascularization of tissue constructs. However, it is not the only point to take into account. Vascularization is a complex process that is highly regulated and depends on many parameters that have to been balanced together. In that recipe, the importance of angiogenesis inductors such as vascular endothelial growth factor family (VEGF), TGF-β, angiopoietin, or fibroblast growth factor (FGF) has to be highlighted. The proportion used for each one and the interaction with inhibitors and other components has to be studied. For example, VEGF-A is the most potent proangiogenic protein described up until now, and FGF stimulates EC proliferation and migration. However, it has been documented that the integrated use of VEGF-A with FGF results in a synergic effect on capillary sprout [117]. Angiotropin has its own role in vascularization, as it induces EC migration. Gene studies on mice have demonstrated that the inactivation of TGF-β caused lethality due to problems with yolk sac vascularization. In addition, the use of a TGF-β antagonist showed decreased blood vessels formation, which enhances its importance. ECM components have also their influence on the vessels’ plexus formation. Heparan sulfate is a proteoglycan that shows interaction with FGFs. Furthermore, FGF has been documented to retain its biological activity when bound to fibrin or fibrinogen, and additionally, it has been shown that it enhances the proliferation activity of EC in vitro when compared to FGF alone [118]. V VWF, apart from its role on coagulation, shows connection with angiogenesis pathways [119]. The interaction between the cell sources used to vascularize has to be pondered. It has been reported that the use of EC with mural cells has as a consequence that the surrounding cells contribute to the stabilization of EC, which has a positive effect on the angiogenesis process. This fact has been studied with smooth muscle cells [120] and pericytes [121]. The influence of each cell type used on vascularization will be analyzed next. However, before that, it is necessary to remark that no standard average rate for cell culturing for vascularization has been established. The development of vascularized substitutes is currently in preclinical progress. For this reason, it is also difficult to determine the ideal size of this kind of emerging substitute based on their future clinical application.

Some publications where the variety of cell sources can be appreciated that have been employed to prevascularize bioengineered skin substitutes are going to be discussed, starting with EC. The use of EC in skin substitutes scaffolds can accelerate blood and lymphatic capillary formation in the dermis both directly after integration to the graft and indirectly by stimulating angiogenesis [122,123]. However, despite EC being used originally, these cells bear the handicap that they do not show high proliferative activity, so they cannot be easily harvested in large quantities beneath clinical conditions [108]. 

Seeing the importance that the prevascularization of the substitute would have, Klar et al. developed a prevascularized substitute in 2014 which included both a dermal and an epidermal layer [116]. The curiosity of this skin equivalent was that the prevascularization was done by using a cell population derived from the stromal vascular fraction (SVF) isolated from human fat. It has been corroborated that cells from the SVF can spontaneously develop a microvasculature either in vitro [124] and in vivo [125,126].

The SVF is a mixed population comprised of a multipotent stem and progenitor cells which include EC, stromal cells, pericytes, and preadipocytes, as well as hematopoietic cells.

Although EC play a vital role in the vascularization process, they are not sufficient to establish a mature vascular network in vitro [127] or in vivo [128]. It has been demonstrated that it is need of the presence of another cell type, commonly mesenchymal, to achieve a mature vascular complex. This promotes the idea of using SVF, which is a combined cell population, instead of one lonely cell type. In addition, as the SVF contains pericytes, these would contribute to vessels remodeling, since pericytes release proangiogenic growth factors that stabilize and lead EC for capillary formation [129].

So, taking the SVF from each patient’s adipose tissue could be a promising autologous approach that avoids immunogenic responses. In addition, this cell population is relatively abundant and accessible in comparison to other adult autologous cell sources.

Klar and her colleagues also showed that particularly SVF cells were a better option for making prevascularized skin substitutes than other EC sources such as human dermal microvascular endothelial cells (HDMEC), human umbilical vein endothelial cells (HUVEC), or human blood outgrowth endothelial cells (HBOEC). For example, when fresh isolates of SVF are used for capillary formation, the ratio of cells that are involved in this process is constant in contrast to when HMDECs are used. When fibroblasts are co-seeded with HDMEC, they can overgrow and dominate the culture, making the cell growth ratio difficult to maintain. However, it is important to notice that the vasculogenic properties of SVF are an exclusive characteristic of fresh SVF isolates. When previously cultured SVF are used, problems such as those mentioned above can be present. As well, it has been documented that HUVEC alone or the combination of HUVEC with fibroblasts did not support microvessel stabilization [116]. Another feature that has to be taken into account when a cell type is elected to prevascularize a substitute is that cell types such as HUVEC and HBOEC are allogenic to the patient, since they derive from sources as umbilical cord and cord blood, which may lead to a non-desired immunogenic response. 

Endothelial progenitor cells (EPCs) have been proposed as a promising alternative for tissue engineering approaches [130]. They have attracted great attention for inducing neovascularization in tissue engineering applications, which would be interesting for clinical applications of autologous cell transplantation. They can be harvested in a minimal-invasive manner from bone marrow or peripheral blood. Although the amount that can be isolated may be low in adults, these cells can be rapidly expanded [131]. Tzyy et al. developed a pre-vascularized 3D-gel scaffold using 250 µl of human plasma in a 24-well culture-plate. EPCs and human fibroblasts were mixed with plasma using various ratios EPC: fibroblast (1:0.5; 1:1, and 1:2). Keratinocytes (1 × 10^6^ cells/cm^2^) were seeded onto an external gel surface [132]. It has been reported that harvesting EPCs in combination with fibroblasts is crucial to the vascular plexus development [133]. They showed that a 3D environment is significant for the formation of a vascularization network, as it provides oxygen supply and nutrient exchange in a way similar to the native skin, which contributes to maintaining the engrafted tissue [134]. When assessing angiogenesis, they found an accumulation of Angiosense 750 fluorophores, a vascularization marker, on the wound and adjacent areas of the prevascularized skin group, which reveals the establishment of a new functional microvasculature. This research group demonstrated that their skin substitute reduced the wound healing process to 7 days after surgery and they showed resembled skin structures 14 days after surgery in a nude mouse healing model.

Adipose-derived stem cells (ASCs) have manifested to hold huge potential to regenerate skin, since they have considerable differentiation plasticity. Chan et al. demonstrated that it is feasible to use the adipose layer of discarded burn skin (dsASCs), even if the patient had an elevated percentage of total body surface area (TBSA) burn to isolate ASCs [135]. This study was performed with military burned patients from the United States of America (USA). The authors proposed the use of dsASCs along with collagen layer scaffolds to develop epithelial and hypodermal layers. To form the epithelial layer, dsASCs were induced with all-trans retinoic acid (ATRA). The dsASCs were induced for an adipocyte differentiation program by supplementing with adipogenic medium to form the hypodermal layer. Simultaneously, to reconstruct a vascularized dermal layer, they seeded dsASCs on a collagen–polyethyleneglycolated–fibrin (PEGylated–fibrin) bilayer hydrogel construct. The dsASCs exhibited fibroblast-like morphology within the collagen layer. In contrast, when they were used within the PEGylated-fibrin layer of the bilayered gel, the dsASCs formed distinct tubular networks and eventually formed dense networks. The positive staining of NG2 indicated that the fibrin-based scaffold supported dsASC differentiation toward a pericyte lineage. These results showed that dsASCs within the bilayer fibrin-based hydrogel may be used as a vascularized dermal equivalent. They finally created a whole full-thickness skin equivalent. In addition, Duttenhoefer et al. developed an in vitro pre-vascularized scaffold using 3D polyurethane as a biomaterial containing nanoparticles of hydroxyapatite. The 9 mm^3^ scaffolds were based on the combination of 7 × 10^4^ EPCs with 7 × 10^4^ ASCs. They indicated the establishment of tubular structures in the scaffolds as early as day 7 in culture [130].

The selection of the right cell source that fits in the scaffold that is going to be used is an essential feature to be balanced. The biomaterials and biopolymer-based scaffolds face the challenge of providing an appropriate microenvironment to maintain the cell proliferation, function, and cell differentiation of each cell population [136]. Three-dimensional scaffolds are emerging to cover this possible gap on the skin equivalents design. By using 3D scaffolds, some cell populations that have been reported as important for prevascularization—for example, the SVF derived from adipose tissue—were cultured efficiently on a scaffold based on a combination of fibrin and collagen. Its three-dimensional structure contributed to the formation of a capillary plexus within the construct, and an efficient revascularization in vivo was achieved. Otherwise, SVF would be lost, because this cell population does not support 2D culture [116]. It has been documented that EC cultured in 3D scaffolds perhaps promote a grafting success rate by improving capillary-like network formation, blood supply as a source of oxygen and nutrients, and lymphatic drainage, which is required for normal skin [137]. So, in conclusion, when 3D hydrogels are used, an adequate environment for the cells’ growth can be supplied, stimulating their proliferation and differentiation as if they were in their native scene [108].

Another interesting strategy since it combines in vitro and in situ prevascularization approaches is the transplantation of microvascular fragments as adipose tissue-derived microvascular fragments (ad-MVF). Ad-MVF are functional vessel segments that rapidly reassemble into microvascular networks after transplantation [138]. This scheme is particularly suitable for intraoperative one-step procedures [139]. Frueh et al. have seeded a dermal matrix with ad-MVF [140] to demonstrate that the prevascularization of Integra^®^ with ad-MVF reinforced the incorporation and epithelization as well as the development of microvascular and lymphatic networks within the matrix [141]. They assessed this experiment on a murine immunocompetent model. They showed a significantly higher oxygenation and vascularization in the preavscularized substitutes when compared to non-seeded controls by the use of a murine immunocompetent model.

Microscale technologies and microfluidic systems also have emerged as a sophisticated in vitro approach for the generation of highly organized microvascular networks [142]. 

To finish, the functionality and long-term survival of in vitro generated immature microvessels differ from that of native blood vessels, independently of the vascularization technique used. To overcome this problem, these features can be improved by gene transfection, but this solution may carry oncogenic risks [143]. An alternative solution may be the co-culture of vascular cells with mural cells as pericytes. Mural cells are essential for the stabilization, maturation, and long-term survival of newly formed microvessels [144,145]. They are also crucially involved in the regulation of vascular permeability, contractile function, coagulation, and immunomodulation [146]. 

## 7. Future Perspectives

Tissue engineering is a promising field that offers solutions that regenerative medicine needs. However, still now, it remains the necessity of improving commercially available skin substitutes to address an efficient wound healing and tissue regeneration. Currently, skin equivalents available on the market are simple and they lack a hypodermal-like layer. An ideal tissue-bioengineered skin substitute for deep wounds would be conformed of three layers that simulates anatomically and functionally all the features of the native skin. Research on this field has to advance to leave behind the stagnation of classic fabrication of epidermal, dermal or composite substitutes in order to fulfill the biological function of bioengineered skin by creating a full-layer prevascularized skin equivalent. This feature is essentially remarkable on deep full thickness wounds where the hypodermal layer is missed and so, vascularization is affected.

In the clinical practice, new needs arise from each clinical case, becoming patient-specific. In an era where personalized medicine is in its apogee, arising bioengineered skin substitutes should be autologous to avoid any risk of rejection. Moreover, future skin substitutes have to achieve an efficient vascularization to provide a better integration with the host tissue and an early wound heal due to a rapid blood supply to the construct.

The translation of cell-based arising skin substitutes to clinical application represents one of the critical challenges on tissue engineering and it has to be overcome with the aim of offering each patient the more efficient therapy that fits with his clinical case and allows him having a good quality of life.

Future perspectives in this field also includes the standardization of the fabrication procedures to minimize the cost of manufacturing of each skin substitutes. The use of bioreactors and bioprinting could achieve this goal by using automatized procedures that would derive on a large-scale fabrication of skin substitutes.

## Figures and Tables

**Table 1:** Classification of temporary skin substitutes ^1^.

Type of Temporary Cover	Example	Description
Xenogeneic decellularized skin	E-Z Derm^®^ Mölnlycke	Porcine xenograft for skin loss injuries
Allogenic cadaveric human skin	Euro Skin Bank	Donated human skin allografts derived from cadavers
Human amnion	EpiBurn^®^ Mimedx	Dehydrated human amnion allograft which acts as a protective barrier and promotes healing
Synthetic dressings	Gauzes or hydrocolloids	Covers made of synthetic materials
Alternative natural skin covers	Banana leaves Potato peel	Natural covers used specially on developing countries

^1^ Types of temporary covers: examples and description.

**Table 2:** Commercially Skin Substitutes ^1^.

Commercial Brand	Cell Content	Source	Conformation	Anatomical Structure	Type of Biomaterial	Description	Clinical Use
AlloDerm^®^ [67]	Acellular	Allogeneic	Bi-layered	Dermal	Biological	Donated allograft human dermis decellularized and freeze-dried with a “dermal” side and a “basement membrane” side	Gingival augmentation, dental roots cover, burns
Apligraf^®^ [45]	Cellular	Allogeneic	Bi-layered	Composite	Biological	Human foreskin neonatal keratinocytes and fibroblasts within a bovine type I collagen matrix	Licensed only for diabetic foot ulcers (DFUs) and venous leg ulcers (VLUs)
Biobrane^®^ [58,68]	Acellular	Xenogeneic	Bi-layered	Dermal	Biosynthetic	Semipermeable silicone film partially imbedded in a 3D network of nylon functionalized with porcine collagen type I	Superficial partial thickness burns
Bioseed-S [69]	Cellular	Autologous	Single-layer	Epidermal	Biological	Autologous keratinocytes suspended on a fibrin sealant	Therapy-resistant chronic VLUs
CryoSkin [47]	Cellular	Allogeneic	Spray	Epidermal	Biological	A cell spray made of keratinocytes isolated from newborn foreskin cultured on silicone	Superficial wounds.
Dermagraft^®^ [48]	Cellular	Allogeneic	Single-layer	Dermal	Biological	Foreskin fibroblast which secrete growth factors and ECM seeded on a bioabsorbable polyglactin mesh scaffold	Stalled diabetic foot ulcers (DFUs), other clinical indications
EPIBASE^®^ [51,70]	Cellular	Autologous	Single-layered	Epidermal	Biological	Keratinocytes isolated from a small biopsy expanded originating CEA that is sprayed on the wound	Cutaneous calciphylaxis, burns
Epicel^®^ [71]	Cellular	Autologous	Single-layered	Epidermal	Biological	Keratinocytes attached to a petrolatum gauze support	Deep dermal burns
EpidexTM^®^ [50]	Cellular	Autologous	Single-layered	Epidermal	Biological	Expanded epidermal keratinocytes precursor cells derived from the follicular outer root sheath (ORS) by plucking hair armed on a sillicone membrane disc	Chronic leg ulcers
GraftJacket^®^ [72]	Acellular	Allogeneic	Single-layered	Dermal	Biosynthetic	Human dermal collagen matrix with vascular channels	Rotator-cuff-tears
Hyalograft 3D^®^ [73]	Cellular	Autologous	Single-layered	Dermal	Biological	Autologous fibroblasts seeded on a hyaluronic acid scaffold	Full-thickness and deep partial wound
Integra^®^ [59]	Acellular	Xenogeneic	Bi-layered	Dermal	Biosynthetic	Matrix of bovine derived collagen fibers, chondroitin-6-sulphate and a silicone sheet that acts as a barrier	Burns or reconstructive surgery
Laserskin^®^ [73]	Cellular	Autologous	Single-layered	Epidermal	Biosynthetic	Cultured keratinocytes on a hyaluronic acid microperforated membrane	Wound resurfacing
Matriderm^®^ [60]	Acellular	Xenogeneic	Single-layered	Dermal	Biosynthetic	A decellularized dermal substitute of bovine origin with collagen matrix coated with α-elastin hydrolysate	For split thickness skin grafting (STSG)
OASIS^®^ [74]	Acellular	Xenogeneic	Single-layered	Composite	Biological	Matrix derived from porcine small intestinal submucosa	Wound closure, full-thickness ulcers
OrCel^®^ [54]	Cellular	Allogeneic	Bi-layered	Composite	Biological	Epidermal keratinocytes and dermal fibroblasts co-cultured in separate layers, into a type I bovine collagen sponge matrix	Severely burned patients
Permacol™ surgical implant [63]	Acellular	Xenogeneic	Single-layered	Dermal	Biological	Decellularized dermal porcine containing collagen and elastin	Specially used for abdominal wall hernia and dermal reconstruction
PolyActive^®^ [75]	Cellular	Autologous	Bi-layered	Composite	Biological	Soft polyethylene oxide terephthalate component and a hard polybutylene terephthalate component with a keratinocytes and fibroblasts	Not specified
Recell^®^ [76]	Cellular	Autologous	Single-layered	Epidermal	Biological	Keratinocytes and melanocytes spray	Depth burns
Suprathel^®^ [64]	Acellular	Cell-free	Single-layered	Epidermal	Synthetic	Porous membrane made of a co-polymer (terpolymer) of poly-dl-lactide, trimethylene carbonate and ε-caprolactone	Partial thickness burns and abrasions
SureDerm^®^ [77]	Acellular	Allogeneic	Bi-layered	Composite	Biosynthetic	Decellularized human dermis coated with gelatin	Exposed orbit after exenteration
Terudermis^®^ [66]	Acellular	Xenogeneic	Bi-layered	Dermal	Biological	Bovine lyophilized cross-linked collagen sponge made of collagen with silicone sheet.	Burns with muscle or bone exposition

^1^ Commercially skin substitutes: cell component, cell source, scaffold conformation, anatomical structure, type of biomaterial and description use.

## Abbreviations

Ad-MVF	Adipose-tissue Derived Microvascular Fragments
AECs	Amniotic Epithelial Cells
AMCs	Amniotic Mesenchymal Cells
ASC	Adipose Stromal Cells
ATRA	All-trans Retinoic Acid
ATMPs	Advanced Therapy Medicinal Products
AV-loop	Arteriovenous Loop
BASS	Bioengineered Artificial Skin Substitutes
BM-MSC	Bone Marrow Mesenchymal Stem Cells
CEA	Cultured Epidermal Autograft
DCs	Dendritic Cells
DFUs	Diabetic Foot Ulcers
dsASCs	Discarded Skin Adipose Stromal Cells
GCP	Good Clinical Practice
GMP	Good Manufacturing Practice
EC	Endothelial Cells
ECM	Extracellular Matrix
EMA	European Medicines Agency
EPCs	Endothelial Progenitor Cells
FDA	Food and Drug Administration
FDCA	Food, Drug and Cosmetic Act
FGF	Fibroblasts Growth Factor
HAM	Human Amniotic Membrane
HBOEC	Human Blood Outgrowth Endothelial Cells
HDMEC	Human Dermal Microvascular Endothelial Cells
HUVEC	Human Umbilical Vein Endothelial Cells
IL	Interleukin
IRB	International Review Board
MHC	Major Histocompatibility Complex
NK	Natural Killer
PEG	Polyethyleneglycol
PHSA	Public Health Services Act
STSG	Split-Thickness Skin Grafts
SVF	Stromal Vascular Fraction
TBSA	Total Body Surface Area
TGF	Transformant Growth Factor
VEGF	Vascular Endothelial Growth Factor
VLUs	Venous Leg Ulcers
